# 

**Published:** 1897-05

**Authors:** 


### CONTENTS—MAY, 1897.—VOL. XII., NO. 11.

ORIGINAL CONTRIBUTIONS—

Report of a Case of Skin Grafting. C. O. Weller, M. D., Austin . . . . . 594
The French Revolutionist, Jean Paul Marat. J. A. Mullen, M. D., Houston 598
Suprapubic Lithotomy. W. F. Chambers, M. D., Austin . . . . . . . . 599
The Triune Man. J. Frank Thornton, M. D., Plum . . . . . . . . . . . 603

SOCIETY NOTES—

New York Academy of Medicine—Section in Orthopædic Surgery . . . 607
South Texas Medical Association . . . . . . . . . . . . . . . . . . . . 609
Fiftieth Anniversary of the American Medical Association . . . . . . . . 610
South Texas Medical Association . . . . . . . . . . . . . . . . . . . . 611
Brazos Valley Medical Association . . . . . . . . . . . . . . . . . . . 612
Southeast Texas Medical Association . . . . . . . . . . . . . . . . . . 614

ABSTRACTS AND SELECTIONS—

"Florid Mendacity." Why Physicians do not Advertise . . . . . . . . 615
Bubonic Plague—Malignant Polyadenitis . . . . . . . . . . . . . . . . 618

EDITORIAL—

Texas State Medical Association . . . . . . . . . . . . . . . . . . . . 629
That Foolish Legislature . . . . . . . . . . . . . . . . . . . . . . . 633
Dr. J. B. Hamilton and "One Water Wyman" . . . . . . . . . . . . . . 635
The Quarantine Question . . . . . . . . . . . . . . . . . . . . . . . 638
A Logical Sequence . . . . . . . . . . . . . . . . . . . . . . . . . . 641
Legislation Run Mad . . . . . . . . . . . . . . . . . . . . . . . . . 643
Family Jars . . . . . . . . . . . . . . . . . . . . . . . . . . . . . 644

MEDICAL NEWS AND MISCELLANY . . . . . . . . . . . . . . . . . 645–651
PUBLISHERS' NOTES . . . . . . . . . . . . . . . . . . . . . . . . 651–652

